# Retinoic Acid Exerts Disease Stage-Dependent Effects on Pristane-Induced Lupus

**DOI:** 10.3389/fimmu.2020.00408

**Published:** 2020-03-20

**Authors:** Leila Abdelhamid, Xavier Cabana-Puig, Brianna Swartwout, Jiyoung Lee, Song Li, Sha Sun, Yaqi Li, A. Catharine Ross, Thomas E. Cecere, Tanya LeRoith, Stephen R. Werre, Haifeng Wang, Christopher M. Reilly, Xin M. Luo

**Affiliations:** ^1^Department of Biomedical Sciences and Pathobiology, Virginia-Maryland College of Veterinary Medicine, Virginia Polytechnic Institute and State University, Blacksburg, VA, United States; ^2^Translational Biology, Medicine and Health Graduate Program, Virginia Polytechnic Institute and State University, Roanoke, VA, United States; ^3^Department of Crop and Soil Environmental Sciences, Virginia Polytechnic Institute and State University, Blacksburg, VA, United States; ^4^Department of Development and Cell Biology, University of California, Irvine, Irvine, CA, United States; ^5^Department of Nutritional Sciences, Pennsylvania State University, University Park, PA, United States; ^6^College of Animal Science, Key Laboratory of Molecular Animal Nutrition, Zhejiang University, Hangzhou, China; ^7^Department of Cell Biology and Physiology, Edward via College of Osteopathic Medicine, Blacksburg, VA, United States

**Keywords:** retinoic acid, pristane-induced, lupus, stage-dependent, kidney, glomerulonephritis, gut microbiota

## Abstract

We previously showed that all-*trans*-retinoic acid (tRA), an active metabolite of vitamin A, exacerbated pre-existing autoimmunity in lupus; however, its effects before the development of autoimmunity are unknown. Here, using a pristane-induced model, we show that tRA exerts differential effects when given at the initiation vs. continuation phase of lupus. Unlike tRA treatment during active disease, pre-pristane treatment with tRA aggravated glomerulonephritis through increasing renal expression of pro-fibrotic protein laminin β1, activating bone marrow conventional dendritic cells (cDCs), and upregulating the interaction of ICAM-1 and LFA-1 in the spleen, indicating an active process of leukocyte activation and trafficking. Transcriptomic analysis revealed that prior to lupus induction, tRA significantly upregulated the expression of genes associated with cDC activation and migration. Post-pristane tRA treatment, on the other hand, did not significantly alter the severity of glomerulonephritis; rather, it exerted immunosuppressive functions of decreasing circulatory and renal deposition of autoantibodies as well as suppressing the renal expression of proinflammatory cytokines and chemokines. Together, these findings suggest that tRA differentially modulate lupus-associated kidney inflammation depending on the time of administration. Interestingly, both pre- and post-pristane treatments with tRA reversed pristane-induced leaky gut and modulated the gut microbiota in a similar fashion, suggesting a gut microbiota-independent mechanism by which tRA affects the initiation vs. continuation phase of lupus.

## Introduction

Systemic lupus erythematosus (SLE) is multi-system autoimmune disorder characterized by breakdown of self-tolerance (1, 2). The hallmarks of SLE include the generation of autoantibodies (3, 4), over-activation of inflammatory cells (5), abnormal pro-inflammatory cytokine storms (6), and the influx of autoreactive cells into different tissues including the kidney, skin, lung, heart, joints, and brain, causing severe collateral tissue damage (1). The disease manifestations are variable due to the complex pathogenesis of SLE (1). With the sometimes paradoxical outcomes of different therapeutics employed in clinical trials (7), understanding the pathogenesis of this disease and the factors contributing to its progression is a necessity for better management strategies of lupus flares.

Vitamin A (VA) has potent immunomodulatory effects that could tone the anti-inflammatory *vs*. pro-inflammatory responses under different contexts, for instance, under steady vs. immunogenic conditions (8). Notably, VA exerts its function through a predominant metabolite known as all-*trans*-retinoic acid (tRA) (9). The effects of tRA treatment in different autoimmune disorders, including type 1 diabetes, multiple sclerosis, and inflammatory bowel disease, were shown to be disease-specific (10). However, in the context of systemic immune dysregulation associated with SLE, VA exerts more complex roles. Even though retinoid treatment in combination with immunosuppressive drugs showed beneficial effects on lupus nephritis (11), previous findings from our group showed that tRA has paradoxical implications on renal inflammation vs. other tissue pathologies. tRA supplementation during active disease aggravated pathologies in non-renal tissues including skin, lungs (12), and brain (13). These findings indicate that tRA may act as an adjuvant to exacerbate the pre-existing, extrarenal inflammation in individuals genetically prone to develop lupus. Consequently, a deeper understanding of its actions is a necessity before clinical recommendations can be made concerning the use of, or avoidance of, VA supplementation in patients with SLE. Therefore, following upon our previous observations, we hypothesize that the effects of tRA on SLE are disease stage-specific. Our current study explores the effects of tRA on the initiation vs. continuation phase of lupus in genetically intact mice. We have used 2,6,10,14-tetramethylpentadecane, or commonly known as pristane, to induce lupus in Balb/c mice. Pristane is a naturally occurring hydrocarbon oil that is known to be the most effective chemical for triggering lupus-specific autoantibodies in mouse models regardless of their genetic background (14). Additionally, this model allows us to control the start of the immunogenic condition and consequently facilitates the investigations of the complex roles of tRA supplementation before and after the induction of lupus. Importantly, pristane-induced lupus confers significant homology to SLE in humans (15, 16).

Here, we show that tRA exerts disease stage-dependent effects on pristane-induced lupus. Pre-pristane tRA treatment exacerbates glomerulonephritis, whereas post-pristane tRA treatment exerts immunosuppressive effects that may dampen lupus-associated kidney inflammation.

## Materials and Methods

### Animals

Three-week-old female Balb/c mice were purchased from The Jackson Laboratory (Bar Harbor, ME) and maintained in a specific pathogen-free environment in a standard 12-h light/dark cycle and in compliance to the Institutional Animal Care and Use Committee (IACUC) at Virginia Polytechnic Institute and State University. All procedures on animals were carried out in accordance with the IACUC approved protocol number 17-123. Mice received hormone-free NIH-31 Modified 6% Mouse/Rat Diet. Food and water were provided *ad libitum*.

### Pristane Injection and tRA Dosing

For the induction of lupus, 3-month-old female Balb/c mice received a single intraperitoneal injection of 500 μl of pristane (Sigma Aldrich), which is known to induce autoantibodies and glomerulonephritis in Balb/c mice to a level similar to that in the classical lupus-prone model, MRL/lpr (17). The mock control group received injection of phosphate-buffered saline (PBS) also at 3 months of age. tRA was purchased from Sigma-Aldrich (St. Louis, MO) and handled in the dark with minimal exposure to air. For oral dosing, tRA was dissolved in canola oil (vehicle), kept in single frozen aliquots, and administered daily. To investigate the effects of tRA on lupus disease and determine if tRA-mediated effects were disease stage-dependent, four groups of mice were established by randomization. The groups included (1) the physiological/mock control that received PBS and vehicle (no pristane, *n* = 8), (2) pristane/vehicle control (*n* = 12), (3) tRA treatment before pristane induction of lupus from weaning (3 weeks) to 3 months of age (*n* = 10), and (4) tRA treatment after pristane induction of lupus from 3 to 9 months of age (*n* = 10). An oral dose of tRA of 1 mg/kg body weight (18) was given daily and adjusted according to body weight obtained biweekly (administered directly to the oral cavity by using a pipette at a volume range of 8–18 μl). This dose was six times lower than the previous dose used by our group (12). Even the higher dose of 6 mg/kg body weight did not show any effect on either serum/liver retinol or liver function enzymes (12). Therefore, we did not expect the lower dose to significantly change these levels. All mice were monitored for 6 months after pristane injection until euthanasia at 9 months of age. At the experimental endpoint, mice were humanely euthanized with CO_2_, followed by exsanguination by transcardiac blood collection according to the IACUC protocol. Total body weight as well as weights of the spleen and both renal lymph nodes (RLNs) were measured, and organ/body weight ratios were calculated. For RNA sequencing analysis of splenocytes, Balb/c mice were treated with tRA at 1 mg/kg body weight from weaning to 3 months of age. Spleens were harvested at 3 months of age and without the injection of pristane.

### Cell Isolation and Flow Cytometry

Isolation of total bone marrow cells and total splenocytes was performed as previously reported (12, 19). Cell pellets were then suspended in 1 ml 1 × red blood cell (RBC) lysis buffer (eBioscience, San Diego, CA) and incubated for 5 min at room temperature, followed by neutralization of the lysis buffer with 5 ml of C10 medium. This solution was further centrifuged and the pellets were resuspended in 5 ml of fresh C10. Our C10 medium is complete RPMI 1640 supplemented with 10% fetal bovine serum, 1 mM sodium pyruvate, 1% 100 MEM non-essential amino acids, 10 mM HEPES, 55 μM 2-mercaptoethanol, 2 mM L-glutamine, and 100 U/ml penicillin–streptomycin (all from Life Technologies, Grand Island, NY). The resulting mononuclear cells were stained for flow cytometry as we reported previously (12). For bone marrow dendritic cell analysis, the following anti-mouse monoclonal antibodies were used: CD11c-APC, CD11b-PE, CD11b-PerCp-Cy5, Siglec-H-PerCP-Cy5.5, I-E/I-A(MHC-II)-FITC (Biolegend, San Diego, CA), and Ly6C-APC-Cy7 (BD Biosciences, San Jose, CA). For analysis of splenic T-cell subsets, we used anti-mouse CD3-APC, CD4-PE-Cy7, CD8-PerCP-Cy5.5, CD44-FITC, and CD62L-APC-Cy7 (Biolegend). Stained cells were analyzed with a BD FACSAria II flow cytometer (BD Biosciences). Flow cytometry data were analyzed with FlowJo.

### Immunohistochemistry

Splenic and kidney sections were embedded in Tissue-Tek OCT Compound (Sakura Finetek) and rapidly frozen in a freezing bath of dry ice and 2-methylbutane. Frozen OCT samples were cryosectioned and unstained slides were stored at −80°C. Immunohistochemical staining procedures were performed as previously described (12, 20). For detection of ICAM1 and LFA1 in the spleen, the following monoclonal antibodies were used: anti-mouse CD3-APC, CD54(ICAM1)-FITC, and CD11/CD18(LFA1)-PE (Biolegend). For detection of renal deposition of IgG, anti-mouse IgG-FITC (eBioscience) was used. Pictures were captured with a Zeiss LSM 880 confocal microscope (Fralin Imaging Center, Virginia Tech). Integrated fluorescence density scores were calculated with the ImageJ software (National Institutes of Health, Rockville, MD).

### Isolation of Intestinal Epithelial Cells (IECs)

After the removal of fat, gut content, and Peyer's patches, the intestine was opened longitudinally, washed in ice-cold PBS, and cut into 1-cm pieces. The pieces were incubated twice in PBS with 5 μM EDTA and 1 μM DTT in a 37°C shaker for 20 min at 200 rpm. Homogenates were intensively vortexed and filtered through 100-μm filters to obtain the IEC-enriched filtrate. IEC suspensions were then centrifuged at 350 × g for 7 min at room temperature. Cell pellets were snap frozen in liquid nitrogen and stored at −80°C for RNA extraction and RT-qPCR.

### RNA Extraction and RT-qPCR

For total RNA extraction, snap-frozen kidney tissues were weighed without allowing them to thaw (whereas snap-frozen IECs were directly processed) and homogenized in Qiazol lysis reagent (Qiagen) using a bullet blender homogenizer (Next Advance, Troy, NY). Total RNA was extracted using RNeasy Plus Universal Kit (Qiagen) that also ensured gDNA elimination. All procedures were performed according to the manufacturers' instructions. Reverse transcription (RT) was performed by using iScript™ Reverse Transcription Supermix (Bio-Rad). Quantitative PCR (qPCR) was performed with PowerUp™ SYBR® Green Master mix and the ABI 7500 Fast Real-Time PCR System (Applied Biosystems). Relative quantities were calculated using the 2–ΔΔCT method. GAPDH and 18S rRNA were used as housekeeping genes for normalization of transcripts from renal and IECs, respectively. Primer sequences for mouse *Tnf*α, *Il1*β, *Il18, Ccl2* (*Mcp1*), *Ccl3* (*Mip1*), *Ccl5, Tgfb*1, *Laminin b1, Itgal*, and *Occludin* and *Claudin-2* tight junction transcripts are available in Table S1. For RNA sequencing, total RNA was extracted from isolated splenocytes using the same method as for IECs.

### RNA Sequencing Analysis

RNA sequencing was performed as previously reported (21). Briefly, all the library preps were performed according to Illumina TruSeq v2 RNA Sample Preparation protocol. Quality of total RNA and cDNA libraries were checked on an Agilent BioAnalyzer 2100 (Agilent Technologies). Samples with RNA integrity scores > 8.0 were selected for RNA-Seq library preps. The cDNA sheared to an average of 300 bp. Individually indexed cDNA libraries were sequenced on one lane of Illumina HiSeq2500 for a minimum of 60 million paired end reads. Following sequencing, data were trimmed for both adaptor and quality using a combination of ea-utils and Btrim (22, 23). Sequencing reads were aligned to the genome (Ensembl.org 38.74) using Bowtie2/Tophat2 (24, 25) and counted via HTSeq (26). Differentially expressed genes (DEGs) were determined using the Benjamini–Hochberg adjusted *P*-value (false discovery rate, or FDR < 0.1) in the R-package DESeq2. Similarly, differential exon usage were tested using DEXSeq (27). Genome expression omnibus GSE56893_RAR_RXR_regions.bed.gz (28) was used to identify tRA receptor binding sites (RAREs). The RNA sequencing data from this study are available in the NCBI SRA database and Gene Expression Omnibus (GEO) repository under accession number GSE140104.

### Microbiota Sampling and Analysis

Fecal samples were obtained at the indicated time points. All samples were stored at −80°C until they are processed. Sample homogenization and cell lysis were carried out via mixing 0.1 g of fecal samples with 0.1-mm sterile zirconia beads and further homogenization was carried out by using the bullet blender homogenizer (Next Advance). DNA extraction was performed using a phenol-chloroform method as previously reported (29). For 16S rRNA sequencing, the V4 region (ca. 252 bp) of 16S rRNA gene was PCR amplified with 515F and 12-base GoLay barcoded 806R primers (30). The purified amplicons were sequenced bidirectionally (paired-end 150 bp) on an Illumina MiSeq at Argonne National Laboratory. 16S rRNA sequencing data were analyzed as described previously (31, 32). The data are available in the NIH SRA database (PRJNA605256).

### Measurements of Renal Function

Weekly urine samples were collected by direct mouse scruff, massaging, and urine collection via a sterile pipette tip at the same time of the day. All samples were stored at −80°C until they are analyzed at the same time with a Pierce Coomassie Protein Assay Kit (Thermo Scientific). At the experimental endpoint, kidneys were harvested, fixed in 10% formalin for 24 h, paraffin embedded, sectioned, and stained with hematoxylin and eosin (H&E) at the Histopathology Laboratory at Virginia–Maryland College of Veterinary Medicine. Slides were read with an Olympus BX43 microscope. Glomerular lesions were graded on a scale of 0–3 for each of the following five categories: mesangial hypercellularity, mesangial matrix expansion, necrosis, the percentage of sclerotic glomeruli, and the presence of glomerular crescents. For categories identifying presence, 0 = absence, 1 = mild, 2 = moderate, and 3 = severe. For categories estimating percentage, 0 = less than 10%, 1 = 10% to 25%, 2 = 25% to 40%, and 3 = 40% or more. At least 50 glomeruli from each kidney were scored. Tubulointerstitial lesions were graded on a scale of 0–3 for each of the following four categories: peritubular mononuclear infiltrates, tubular damage, interstitial fibrosis, and vasculitis. All scores were graded in a blinded fashion by a certified veterinary pathologist (Dr. Cecere) based on a global semi-quantitative assessment as previously reported (33).

### ELISAs and Quantification of Endotoxin

Serum samples were obtained following whole blood collection and coagulation, and stored at −20°C. Anti-double stranded (ds)DNA IgG levels in diluted serum samples was detected following a previously reported ELISA assay (12). Briefly, the plate was coated with 0.1 mg/ml of calf thymus DNA (Sigma) in 1× saline-sodium citrate buffer and incubated at 4°C overnight, followed by washing with PBS containing 0.05% Tween-20 (PBS-T). Wells were then blocked with PBS containing 1% BSA for 1 h at room temperature. Samples were added and incubated for 1 h at room temperature. After additional washes in PBS-T, horseradish peroxidase-conjugated goat anti-mouse IgG-Fc secondary antibody (Bethyl Laboratories, Montgomery, TX) was added and incubated for 1 h at room temperature, followed by further washing. 3,3′,5,5′-Tetramethylbenzidine (TMB) substrate (Biolegend) was then added, and the reaction was stopped by adding 2N H_2_SO_4_ stop solution. The plate was read at 450 nm using SpectraMax plate reader (Molecular Devices, Sunnyvale, CA). Total IgG levels were determined using a mouse IgG ELISA kit (Bethyl Laboratories) according to the manufacturer's instructions. Serum endotoxin (lipopolysaccharide, or LPS) level was measured using Pierce LAL Chromogenic Endotoxin Quantitation Kit (Thermo Scientific) following the manufacturer's procedures.

### Quantification of Retinol/VA

For serum retinol/VA quantification, an aliquot (45–60 μl) of serum sample was pipetted in 100% pure ethanol, and incubated at room temperature for an hour. Then, samples were saponified during a 30-min water bath process after addition of pyrogallol and potassium hydroxide. Hexanes and water were then added to each sample after saponification for retinol extraction. After sufficient vortexing, samples were centrifuged, an aliquot of hexane upper phase was transferred into a vial, and a known amount of trimethylmethoxyphenyl-retinol (TMMP), serving as internal standard, was added to each vial. The hexane extract in each vial was dried under a stream of nitrogen and reconstituted in 100 μl methanol for Ultra Performance Liquid Chromatography (UPLC) analysis. A C-18 column, with methanol:water (92.5:7.5, 0.6 ml/min) used as the mobile phase, was applied in the UPLC system, while peaks were detected by UV absorbance at 325 nm. Retinol mass was determined based on the ratio of the integrated areas of the TMMP and retinol peaks, and the known amount of the added TMMP standard. For analysis of tissue VA content, 0.05 g of frozen liver samples were weighed and homogenized with 100% pure ethanol. The subsequent steps were the same as described in the serum analysis above.

### Statistical Analysis

One-way ANOVA followed by Dunnett's posttest was employed for the comparisons between tRA-treated groups and the pristane-only control. For analysis involving the physiological/mock control (no pristane), one-way ANOVA followed by Tukey's posttest was performed. Data are shown as mean ± standard error of the mean. Significant differences were shown as ^*^P < 0.05, ^**^P < 0.01, ^***^P < 0.001, ^****^P < 0.0001. Spearman correlation analysis was performed to determine correlations between immunological changes and the disease phenotype. All analyses were performed with Prism GraphPad.

## Results

### Progressive Glomerulonephritis With tRA Pre-treatment in Pristane-Induced Lupus

tRA treatment of lupus-prone MRL/lpr mice showed paradoxical effects on renal inflammation vs. other tissue pathologies including inflammation of the skin, lungs (12), and neuroinflammation (13), suggesting that tRA can expand the pre-existing immunogenic environment in extrarenal organs in genetically prone lupus. In this study, we further hypothesized that the effects of tRA on lupus disease were disease stage-dependent. We explored the roles of tRA during different stages of the disease, namely, before and after the initiation of lupus using the pristane-induced lupus model. Compared to mock-control (PBS-treated) Balb/c mice, a single intraperitoneal injection of pristane induced significant splenomegaly in all mice (Figure S1A) and detectable enlargement of RLNs in 42% of the treated mice (Figure S1B). Pristane-treated mice also showed significantly reduced body weights (Figure S1C) with significant reduction of serum (Figure S1D) and liver (Figure S1E) retinol/VA levels. Pristane-induced renal inflammation was limited to the glomeruli that could be classified as ISN/RPS Class II—mesangial proliferative lupus nephritis (34). This was indicated by significantly increased overall glomerular scores shown as the sum of mesangial hypercellularity and mesangial matrix formation scores (Figure S1F) with no significant changes in tubulointerstitial lesions (Figure S1G).

**Figure 1:**
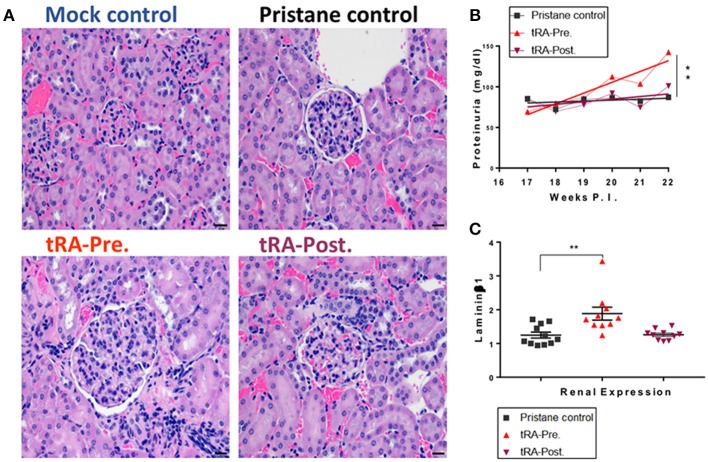
Progressive glomerulonephritis with tRA pre-treatment in pristane-induced lupus. SLE-like disease was assessed 6 months after pristane injection and upon oral administration of tRA either before (tRA-Pre.) or after (tRA-Post.) pristane induction of lupus (*n* ≥ 10 mice per group). **(A)** Representative micrographs for H&E-stained kidney sections at 20×. **(B)** Levels of proteinuria over time. Linear regression analysis was employed to determine the slope deviation from zero. **(C)** Relative mRNA expression of matrix protein Laminin β1 in the kidney after normalization to that of GAPDH. Significant differences were shown as **p < 0.01.

Oral doses of tRA (1 mg/kg body weight) vs. vehicle control were given daily, from 3 weeks of age to the time of lupus induction at 3 months (tRA pre-treatment), or from 3 months to the experimental endpoint at 6 months post lupus induction (tRA post-treatment). Whereas no significant difference was found to distinguish pristane/tRA-treated groups from pristane alone (Figure 1A), severe renal lesions were only observed in tRA-pre-treated mice, where 20% of mice showed glomerular scores of 5 (the sum of mesangial matrix expansion and hypercellularity scores). Consistent with this, compared to mild proteinuria levels in pristane alone or pristane/tRA-post-treated mice, the pre-treatment of tRA aggravated the severity of glomerulonephritis with significantly increased proteinuria levels over pristane alone (Figure 1B). Moreover, the combination of pristane and pre-treatment with tRA induced detectable enlargement of RLNs in 50% of pre-treated mice compared to only 20% in post-treated mice (Figure S1B). Importantly, compared to the pristane control, only the tRA pre-treatment showed a significant increase in the renal expression of the pro-fibrotic molecule laminin β1 extracellular matrix protein transcript (Figure 1C), which is known to contribute to mesangial proliferative nephritis (35). Meanwhile, an upregulated renal expression of TGFβ1, a major pro-fibrotic mediator (36, 37), was observed with pristane alone and the combination of pristane and pre-treatment with tRA compared to the physiological/mock control (Figure S1H). These findings suggest that tRA supplementation before the establishment of immunogenic changes of lupus could aggravate the initiation of glomerulonephritis while the post-treatment with tRA did not significantly change the renal disease induced by pristane.

### Enhancement of Systemic Inflammation by tRA Pre-treatment in Pristane-Induced Lupus

**Figure 2:**
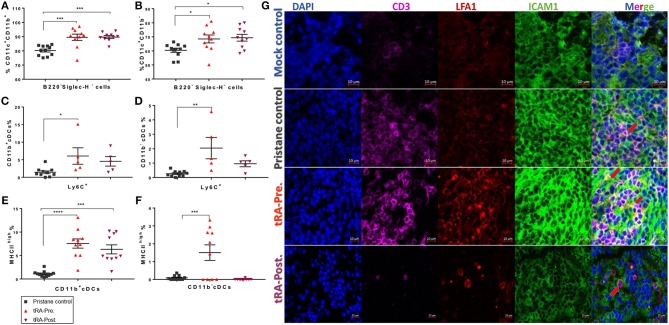
Enhancement of systemic inflammation by tRA pre-treatment in pristane-induced lupus. **(A,B)** The percentages of bone marrow cDC subsets determined by flow cytometry at the experimental endpoint (6 months post induction of lupus) are shown. Expansion of CD11b^+^ **(A)** and CD11b^−^ **(B)** cDCs. **(C,D)** Upregulation of the inflammatory marker Ly6C on CD11b^+^ **(C)** and CD11b^−^ **(D)** subsets of cDCs. **(E,F)** Percentage of MHC-II^high^ cells on CD11b^+^ **(E)** and CD11b^−^ **(F)** cDCs. **(G)** Immunohistochemical stains of splenic sections showing the infiltration of T cells (CD3, purple) and the expression of CD11/CD18 (or LFA1, red) and CD54 (or ICAM1, green). Pictures were captured with a Zeiss LSM 880 confocal microscope (red arrows indicate the interaction between LFA1 and ICAM1). Significant differences were shown as *p < 0.05, **p < 0.01, ***p < 0.001, ****p < 0.0001.

To better understand the mechanism by which the pre-treatment with tRA, but not the post-treatment, exacerbated pristane-induced glomerulonephritis, we investigated how tRA treatments at different disease stages affected immune cell populations in lymphoid organs. Cellular regulation is critical for the pathogenesis of chronic inflammation in pristane-induced lupus (38, 39). tRA is known to paradoxically modulate immune cells to either sustain homeostasis under steady states (40) or promote inflammatory responses in an immunogenic environment (41). Flow cytometry staining of bone marrow cells showed that compared to pristane alone, tRA supplementation either pre- or post-induction of lupus significantly induced the expansion of CD11b^+^ (Figure 2A) and CD11b^−^ (Figure 2B) subsets of conventional dendritic cells (cDCs) gated as B220^−^ Siglec-H^−^ in CD11c^+^ myeloid cells. However, for the inflammatory marker Ly6C, only tRA pre-treatment, but not tRA post-treatment, significantly induced the inflammatory phenotype of CD11b^+^ and CD11b^−^ cDCs compared to pristane alone (Figures 2C,D). Similarly, tRA pre-treatment significantly upregulated the activation marker MHC-II on both cDC subsets compared to pristane alone, whereas the post-treatment only increased MHC-II expression on CD11b^+^ cDCs (Figures 2E,F). This suggests that the combination of pristane and tRA pre-treatment may target cDCs in the bone marrow to facilitate glomerulonephritis. This notion is supported by our previous report that activated cDCs from the bone marrow can migrate to the kidney to promote glomerulonephritis in MRL/lpr mice (42). Interestingly, both tRA pre- and post-treatments upon pristane injection significantly expanded the monocytic myeloid-derived suppressor cells (MDSCs) gated as Ly6C^low/intermediate^ CD11b^+^CD11c^−^ compared to pristane alone (Figure S2A). In contrast, a significant reduction of plasmacytoid DCs (pDCs), another subset of DCs known to promote the pristane-induced systemic inflammation (43), was observed for both tRA pre- and post-treatments (Figures S2B,C). This suggests that neither MDSCs nor pDCs may be involved in the differential disease phenotypes seen with tRA pre-treatment vs. tRA post-treatment.

We also analyzed the spleen and observed a reduced percentage of CD62L^+^CD44^−^ naïve T cells along with an increased percentage of CD62L^−^CD44^+^ effector memory T cells (T_EM_ cells) in the tRA pre-treated group (Figure S2D), which were trending but not statistically significant. This suggests a potential effect of tRA pre-treatment in promoting splenic T-cell activation. Notably, a possible interaction between intercellular adhesion molecule 1 (ICAM1)-expressing endothelial cells and leukocyte function-associated antigen 1 (LFA1)-expressing lymphocytes was observed for the tRA pre-treated group in immunohistochemically stained splenic sections (Figure 2G and Figure S2E), indicating an active process of leukocyte activation and trafficking. These findings suggest that pre-treatment with tRA can enhance pristane-induced systemic inflammation by activating cDCs in the bone marrow and T cells in the spleen. These activated cells may migrate to the kidney and contribute to renal inflammation leading to aggravated glomerulonephritis.

### DEGs Induced by tRA Pre-treatment

**Figure 3:**
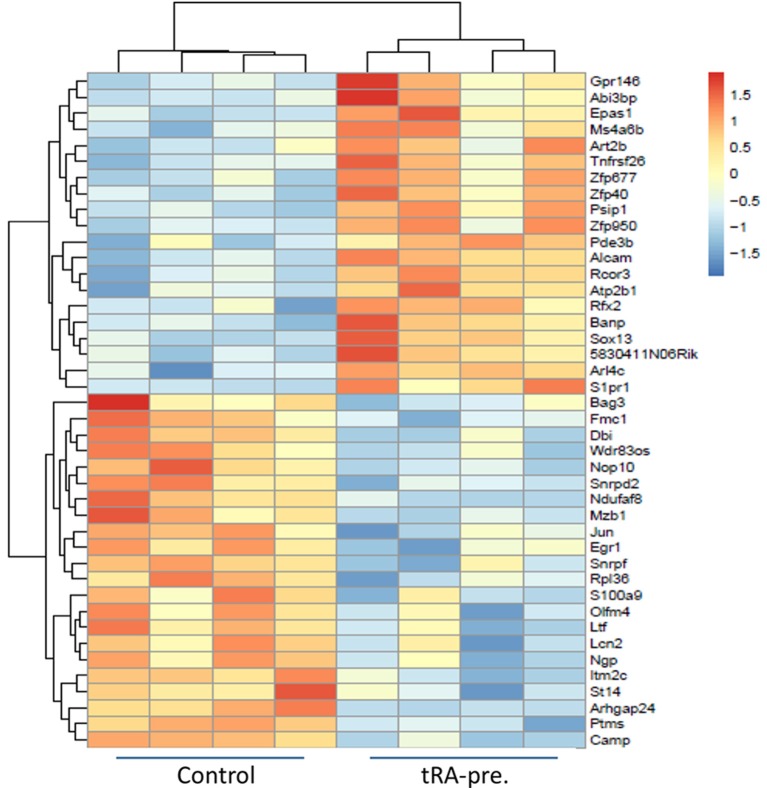
Differentially expressed genes induced by tRA pre-treatment. RNA sequencing (*n* = 4 mice per group) was performed on total splenocytes harvested from Balb/c mice at 3 months of age following daily oral dosing of vehicle or tRA (no pristane). A heatmap with a cutoff FDR of <0.1 is shown. Red, upregulation; blue, downregulation.

tRA modulates the differentiation and functional decisions of immune cells through transcriptional regulation (44, 45). Since the adverse effect of tRA pre-treatment on renal inflammation may be explained by the induction of activated cDCs (Figure 2) that we had previously shown to migrate to the kidney to provoke renal pathology (42), we were interested in identifying candidate genes and transcripts induced by tRA that were associated with cDC activation. Balb/c mice received oral tRA from weaning till euthanasia at 3 months of age without further injection of pristane. RNA sequencing was performed on the total splenocytes harvested from these mice. Our chosen dose of tRA (1 mg/kg body weight) changed the expression of 42 coding genes with an FDR of <0.1 (Figure 3). Of these DEGs, 20 were upregulated and 22 were downregulated. When the FDR cutoff was set at 0.05, only 12 upregulated DEGs and 6 downregulated DEGs were identified. All these genes were found to possess at least one putative RARE in their promoters (Table S2), indicating that they are direct targets of tRA-mediated regulation (46).

The observed changes on the transcript level provided further explanation to the inflammatory changes that we noted with tRA pre-treatment. Based on the transcriptomic signature, we found that our splenic cell populations were similar to CD5^−^CD163^+^ inflammatory cDC2, a population that was shown by high-dimensional single-cell protein and RNA expression analyses to expand in human SLE patients (47). Among the significantly upregulated DEGs following tRA treatment, we identified endothelial PAS domain-containing protein 1 (*Epas1*) (48) and ADP ribosylation factor like GTPase 4C (*Arl4c*) (49) that were associated with enhanced functional potential of cDC2 (47). In particular, *Epas1* is a known regulator of IL-31 (50) and IL-31 signaling in activated cDCs augments their inflammatory potential (51).

Other upregulated DEGs in our study included activated leukocyte cell adhesion molecule (*Alcam*) and a G protein-coupled receptor gene, sphingosine-1-phosphate receptor 1 (*S1pr1*). *Alcam* is required for cDC migration via afferent lymphatics, and its blockade through high-affinity antibodies induces cDC retention and alleviates cDC-induced allogenic responses (52). Further, since migratory cDCs can activate T cells through a stable immunological synapse (53), the upregulated *Alcam* level may explain the increase of T_EM_ cells vs. naïve T-cell populations following pre-treatment with tRA. *S1pr1*, on the other hand, is involved in both cDC functions and autoimmunity. The interaction between S1PR1 and its ligand S1P is known to regulate cDC activities (54), where the downstream signaling modulates various cDC functions including antigen capture, cytokine production (55), and cDC migration (56, 57). Importantly, this interaction has been shown to contribute to the pathogenesis of autoimmune diseases (58). Strategies that target this signaling pathway have been shown to ameliorate not only nephritis (59) but also systemic autoimmunity in lupus mice (60).

Collectively, these results suggest that pre-treatment with tRA potentiate pristane-induced immunogenic changes through transcriptional regulation of multiple genes (e.g., *Epas1, Arl4c, Alcam, S1pr1*) involved in cDC activation and migration, which have been shown to greatly contribute to the pathogenic role of cDCs in glomerulonephritis (61–63).

### Immunosuppressive Functions of tRA Post-treatment in Pristane-Induced Lupus

**Figure 4:**
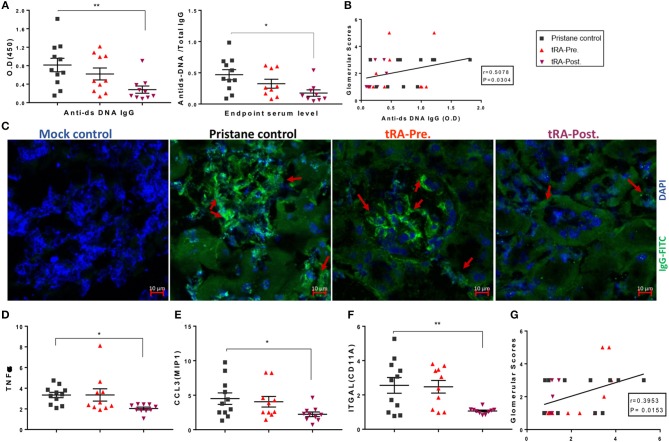
Immunosuppressive functions of tRA post-treatment in pristane-induced lupus. **(A)** Serum levels of anti-dsDNA IgG antibodies and their ratios to total IgG antibodies at 6 months post pristane injection as determined with ELISA. **(B)** Correlation between autoantibody levels and glomerular pathological scores as determined with Spearman correlation analysis. **(C)** IgG deposition in the kidney as determined with immunohistochemical staining using an anti-IgG monoclonal antibody (green) and DAPI (blue). Red arrows indicate IgG deposition. **(D–F)** Renal transcript levels of *Tnf*α **(D)**, *Ccl3*/*Mip1* **(E)**, and *Itgal* subunit of LFA1 **(F)** as determined with RT-qPCR. **(G)** Correlation between renal *Itgal* expression and glomerular pathological scores. Significant differences were shown as *p < 0.05, **p < 0.01.

While tRA supplementation after pristane induction did not change the severity of renal disease in our study (Figure 1), previous studies from other investigators had shown beneficial effects of tRA after the onset of glomerulonephritis (33, 64). We thus expanded our investigation to determine whether tRA post-treatment could play an immunosuppressive role in pristane-induced lupus. Since circulatory autoantibodies could provoke renal pathology through their deposition into the kidney (65), we tested serum autoantibody levels and renal deposition of IgG. Consistent with previous reports where tRA supplementation during active disease was protective in genetically prone lupus nephritis mouse models, we detected a significantly lower level of anti-dsDNA autoantibodies in the serum as well as its ratio to total IgG with tRA post-treatment compared to pristane alone (Figure 4A). There was a moderate correlation between autoantibody levels and glomerular pathology (Figure 4B), suggesting that a lower level of circulatory autoantibodies with tRA post-treatment may have benefited renal health even though the disease phenotype was unchanged. In support of this, we observed a moderate reduction of IgG deposition in the kidney with tRA post-treatment (Figure 4C and Figure S3).

Aberrant regulation of different cytokines could lead to abnormal cell activation, tissue infiltration of activated cells, and subsequent collateral damage associated with SLE (6, 66). Therefore, we determined the renal expression of several key pro-inflammatory cytokines and chemokines known for their roles in modulating leukocyte activation and migration. Compared to the mock control (data not shown), we found that in the renal tissue, both pristane alone and the combination of pristane and tRA pre-treatment upregulated a number of pro-inflammatory cytokines, whereas the combination of pristane and post-treatment with tRA downregulated the tested pro-inflammatory transcripts to levels similar to the physiological control. More importantly, compared to the pristane-alone control, significant reductions in the renal expression of TNFα (Figure 4D) and macrophage inflammatory protein 1-alpha (or CCL3; Figure 4E) were observed following tRA post-treatment but not the pre-treatment. The level of renal TNFα, in particular, was positively correlated with glomerular pathology (Figure S4A), suggesting that the downregulation of renal TNFα with tRA post-treatment might have reduced glomerular damage. In addition, we observed trending reductions in the levels of monocyte chemoattractant protein 1 (or CCL2; Figure S4B), RANTES (or CCL5; Figure S4C), and inflammatory cytokines of IL-1 family including IL-18 (Figure S4D) and IL-1β (Figures S4E,F) with tRA post-treatment. In contrast, these levels were slightly increased with tRA pre-treatment. Moreover, pristane-induced upregulation of the renal expression of the alpha subunit of LFA1, integrin αL (ITGAL), was significantly reduced with tRA post-treatment compared to pristane alone (Figure 4F). The renal level of ITGAL was positively correlated with glomerular pathology (Figure 4G), suggesting that tRA post-treatment may have protected against glomerulonephritis even though the benefit was not obvious. Together, these results suggest that tRA treatment after the onset of glomerulonephritis exert immunosuppressive functions as previously reported (33, 64).

### tRA-Induced Microbiota Changes in Pristane-Induced Lupus

**Figure 5:**
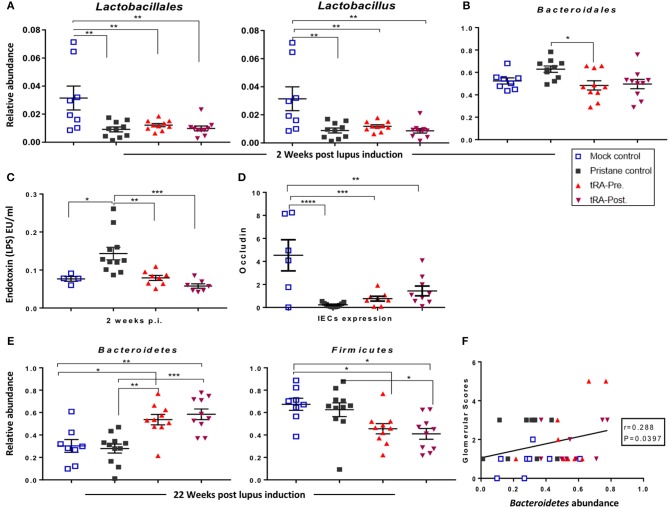
tRA-induced microbiota changes in pristane-induced lupus. **(A)** Quantitation of the relative abundance of the order *Lactobacillales* and genus *Lactobacillus* at 2 weeks following lupus induction as determined with 16S rRNA sequencing. **(B)** Relative abundance of *Bacteroidales* at the same time point. **(C)** Serum endotoxin levels at the same time point. **(D)** Expression of tight junction transcript *Occludin* in the IECs at 6 months post pristane injection. **(E)** Relative abundance of the phyla *Bacteroidetes* and *Firmicutes* at 22 weeks post lupus induction. **(F)** Correlation between the relative abundance of *Bacteroidetes* and glomerular pathological scores. Significant differences were shown as *p < 0.05, **p < 0.01, ***p < 0.001, ****p < 0.0001.

The change of gut microbiota could be a driving force for SLE especially under genetically prone conditions (20, 31, 67). However, whether gut dysbiosis could develop in environmentally driven lupus like the pristane-induced model and whether it would contribute to lupus pathogenesis in this model remain unclear. Therefore, we utilized 16S rRNA sequencing to characterize the changes of gut microbiota following pristane injection and upon the supplementation of tRA at different stages of the disease. Compared to the physiological/mock control, as early as 2 weeks post lupus induction, pristane injection triggered a significant reduction of the relative abundance of beneficial bacteria in the order *Lactobacillales*, genus *Lactobacillus* (Figure 5A), and on the species level, a reduced relative abundance of *Lactobacillus gasseri* (Table S3). Meanwhile, the injection of pristane transiently induced a significant increase of the order *Bacterodiales* at 2 weeks (Figure 5B) and significantly enriched the relative abundance of the genus *Ruminococcus* at 4 weeks post pristane injection (Figure S5A). More importantly, pristane injection significantly increased serum endotoxin levels at 2 weeks post pristane injection (Figure 5C). Similarly, it significantly reduced the transcript level of the barrier-forming tight junction protein *Occludin* (Figure 5D) and induced a moderate increase in the expression of the pore-forming tight junction protein transcript claudin-2 (*Cldn2*) (Figure S5B) in the IECs as observed at 6 months post induction. These observations indicate that pristane was able to recapitulate a phenotype similar to a leaky gut described in genetically prone models of SLE (10, 20), further supporting the leaky gut as a danger signal for autoimmunity even in the absence of genetic predisposition.

Interestingly, at the early stage of lupus initiation (2 weeks post pristane injection), both tRA treatments significantly reduced the serum endotoxin levels that was induced by pristane (Figure 5C). At 6 months post pristane injection, both tRA treatments moderately increased the IEC expression of *Occludin* compared to pristane alone (Figure 5D). In addition, at 2 weeks post pristane injection, both tRA treatments significantly increased the relative abundance of the order *Clostridiales* and genus *Clostridium* (Figure S5C) over pristane alone and modulated the abundance of several bacterial species (summarized in Table S3). Moreover, starting from 10 weeks post pristane injection, both tRA treatments significantly increased the relative abundance of *Bacteroidales* (Figure S5D) over both the mock-control and pristane-alone groups. Furthermore, compared to pristane alone, both tRA treatments significantly enriched the abundance of *Bacteriodetes* while reducing the abundance of *Firmicutes* (Figure 5E) as well as the *Firmicutes/Bacteriodetes* ratio (Figure S5E), although only the effects of tRA post-treatment were statistically significant. Notably, the abundance of *Bacteroidete*s was positively correlated with glomerular pathological scores (Figure 5F). Collectively, these observations suggest that tRA may be able to reverse pristane-induced leaky gut but appear to modulate pristane-induced microbiota changes regardless of the time of administration.

## Discussion

Following upon the previous findings by our group that tRA acted as an adjuvant to exacerbate the pre-existing inflammation in extrarenal tissues in mice genetically prone to develop lupus (12), we investigated the hypothesis that the effects of tRA on SLE could be disease stage-specific. Here we utilized the pristane model to induce lupus in Balb/c mice (14). This model allowed us to control the timing of immunogenic changes, enabling us to investigate the immunomodulatory effects induced by tRA given before (tRA pre-treatment) vs. after (tRA post-treatment) the induction of lupus.

Pristane induces the accumulation of autoantigens and the production of various autoantibodies resulting in immune dysregulation resembling that in human SLE. It is an effective chemical to induce glomerulonephritis in many murine models (14, 15, 17). Pristane injection in our study increased the level of circulating autoantibodies as well as the deposition of the IgG in the kidney. As anticipated, a positive correlation was found between the elevated anti-dsDNA level and the glomerular pathology in agreement with what is known for human SLE (68). Interestingly, however, the glomerular pathology was only apparent (with a composite glomerular inflammatory score of 5) and associated with significantly elevated proteinuria levels, which indicate renal damage, when mice were treated with tRA before pristane induction of lupus. In contrast, the supplementation of tRA after lupus induction did not change the severity of pristane-induced glomerulonephritis. This observation was not contradictory to our previous findings in genetically lupus-prone MRL/lpr mice, where tRA treatment during the progressive phase of the disease ameliorated glomerulonephritis (12). In fact, we went on to show that compared to pristane alone, tRA post-treatment possessed immunosuppressive functions on autoantibody levels and renal expression of pro-inflammatory cytokines/chemokines, which is in agreement with previous reports in MRL/lpr (33) and NZB/W F1 (64) lupus-prone mice.

Lupus nephritis affects about 50 to 60% of SLE patients and could be fatal if left without an effective treatment (69, 70). Glomerulonephritis could result from increased proliferation of mesangial cells (the cells forming the glomerulus) and accumulation of extracellular matrix (ECM) that could lead to tissue scarring and loss of renal functionality (71). Our data suggest that the pre-treatment with tRA may accelerate the initiation of pristane-induced mesangial proliferative glomerulonephritis through directly enhancing the proliferation and activity of mesangial cells. Retinoids including tRA generally promote the proliferation of mammalian cells (72). In particular, tRA has a potent anti-apoptotic potential on mesangial cells (73). Indeed, we observed mesangial hypercellularity in the glomeruli and expansion of ECM following pristane injection. Interestingly, over pristane alone, only the pre-treatment with tRA significantly augmented the renal expression of laminin β1, a major ECM protein produced by mesangial cells that has a known pathogenic role in proliferative nephritis (35). In addition, we observed a significant increase in the expression of renal TGFβ1 in tRA-pre-treated mice but not the post-treated group. TGFβ1 signaling is associated with glomerulopathy (74) where TGFβ1 could induce the production and aberrant deposition of different laminins (including laminin β1) on the glomerular basement membrane (GBM) as an initial signal for pro-fibrosis stages of lupus nephritis (37). In particular, laminin β1 acts as a nucleosome ligand where its expression in GBM and binding to the nucleosomes could help provide antigens for autoantibodies and represent a nidus for the initiation of lupus nephritis (37). Consistent with this, we showed increased deposition of total IgG in the kidney following pristane injection and tRA pre-treatment, suggesting the initiation of these pro-fibrotic signals.

The pathogenesis of pristane-induced chronic inflammation depends on continuous influx of leukocytes including cDCs and lymphocytes to the inflamed peritoneal cavity and peripheral tissues (75, 76). The recruitment of immune cells is tightly regulated by the surrounding cytokine milieu (38, 39). Similarly, tRA is known to modulate the homing capacities of different immune cells (40) and to modulate immune cell activation and functions (77). Therefore, we examined the transcript levels of inflammatory cytokines and chemokines known for their roles in lupus development and progression (78, 79) as well as the cellular changes upon pristane injection and tRA supplementation in this model. Our findings suggest that pre-treatment with tRA could facilitate the immunogenic events in pristane-induced lupus through direct modulation of immune cell activation and trafficking to the splenic and renal compartments. Interestingly, we observed an interaction between ICAM1- and LFA1-expressing cells in the spleen that was potentiated by the combination of pristane and tRA pre-treatment. This interaction suggests the presence of a costimulatory signal for an active process of leukocyte activation and trafficking (80, 81).

In contrast, tRA treatment after pristane induction of lupus did not exacerbate glomerulonephritis. The differential effects of tRA pre-treatment vs. post-treatment may be due to enhanced activation of cDCs following pre-treatment with tRA. Although both pre- and post-pristane tRA treatments induced the expansion of bone marrow cDCs, only tRA pre-treatment upregulated the expression of Ly6C. Similarly, only the pre-treatment induced MHC-II expression on CD11b^−^ cDCs over pristane alone. tRA is known to regulate the activities of antigen-presenting cells including cDCs and macrophages (44, 82). Activation of cDCs is required for the induction of glomerular injury and the progression of renal immunopathology in murine models (61, 62). Infiltration of activated cDCs into the kidney aggravates the progression of chronic inflammation associated with chronic kidney damage associated with lupus (42, 63). Therefore, the results in this study suggest that tRA pre-treatment may have facilitated antigen presentation of apoptotic products—which are abundant following pristane injection (83)—by MHC-II^high^ cDCs, which subsequently amplified systemic and renal inflammation leading to exacerbated glomerulonephritis. Moreover, the activated cDCs with tRA pre-treatment may have directly contributed to the activation of mesangial cells in the kidney through producing an array of pro-inflammatory cytokines and chemokines (84–88). Recent findings by Yu et al. (89) provide support for our explanation that the potentiation of mesangial cell activity by tRA pre-treatment contributes to exacerbation of pristane-induced glomerulonephritis. They found that *in vitro*-activated mesangial cells could act as nonprofessional antigen-presenting cells and express cell surface molecules associated with antigen presentation including MHC-II, ICAM-1, CD40, and CD80, and therefore were able to prime co-cultured T cells to promote their activation and polarization toward T helper-1 (Th1) cells (89). It is likely that the combination of pristane and tRA pre-treatment creates a favorable cytokine/chemokine environment for mesangial cell proliferation, whereas the proliferating mesangial cells would acquire abilities to present antigens, thereby promoting Th1 differentiation and renal inflammation.

tRA post-treatment, on the other hand, did not further induce cDC activation over pristane alone. Instead, it significantly reduced renal expression of pro-inflammatory cytokines and chemokines, thus reducing mesangial cell activation and renal inflammation. In addition, it significantly decreased the serum level of autoantibodies and reduced IgG deposition in the kidney, which also dampen renal inflammation. Taken together, our findings suggest that the interplay between the activation of cDCs and mesangial cells may explain the disease stage-dependent effects of tRA on pristane-induced renal damage.

Leaky gut and microbial dysbiosis provide danger signals for the development of autoimmunity (10, 20). Although germ-free mice also developed pristane-induced lupus (90), the presence of microbiota may augment nephritis in this model. Mice housed under specific pathogen-free conditions have significantly ameliorated autoantibody levels compared to conventionally housed mice following pristane injection (91). Pristane has been shown to induce aberrant expression of pathogen recognition receptors, namely, toll-like receptors (TLRs) including TLR4 and TLR9, in the kidney, and these TLRs are necessary for the development of pristane-induced nephritis (92). Bacterial component and TLR4 ligand LPS (93), present in the gut microbiota, can aggravate the inflammatory activities of peritoneal cells isolated from pristane-treated mice (90). In addition, a recent report has shown that a leaky gut induced by dextran sulfate sodium can augment autoimmunity in pristane-induced lupus (94). However, to our knowledge, no previous studies have shown whether gut dysbiosis is present in the pristane model and whether it would contribute to its pathogenesis. In this study, pristane injection significantly modulated the relative abundance of beneficial bacteria in the order *Lactobacillales*, genus *Lactobacillus* as early as 2 weeks post injection. In addition, it transiently induced a significant increase of the order *Bacterodiales* and significantly enriched the relative abundance of the genus *Ruminococcus*. It is noteworthy that reduced *Lactobacillales* has been associated with the progression of glomerulonephritis (31) and that enriching their abundance through oral gavage significantly ameliorates disease progression in MRL/lpr mice (20). Since *Lactobacilli* positively regulate the intestinal epithelial barrier functions (95, 96) and that their enrichment can reverse the leaky gut in MRL/lpr mice (20), we tested the level of serum endotoxin and expression of intestinal tight junction transcripts in the current study. Strikingly, a leaky gut was also present in pristane-induced lupus. This suggests that the pristane model could recapitulate the gut dysbiosis associated with SLE. Also, since changes in gut microbiota and the observed signs of a leaky gut occurred right after pristane injection and before the onset of obvious disease, our findings further support the leaky gut as a danger signal for autoimmunity even in the absence of genetic predisposition (10, 20).

Both tRA treatments further modulated the microbiota changes noted in the pristane-induced lupus model. At early stage of lupus initiation, both tRA treatments significantly reduced the serum endotoxin levels induced by pristane. Additionally, the combination of pristane and tRA treatments significantly increased the expression of *Occludin* over pristane alone. Strikingly, both tRA treatments upon pristane injection significantly increased the relative abundance of the order *Clostridiales* and genus *Clostridium* and modulated the abundance of several bacterial species and consequently reversed the microbiota dysbiosis previously described in human SLE (67). However, both tRA treatments combined with pristane significantly increased the relative abundance of *Bacteroidales* and enriched the abundance of *Bacteriodetes* while reducing *Firmicutes*, consequently reducing the *Firmicutes/Bacteriodetes* ratio. This is similar to the reduced *Firmicutes/Bacteroidetes* ratio in SLE patients compared to healthy controls (97), suggesting that tRA treatments may contribute to lupus pathogenesis. Consistent with this notion, the increased abundance of *Bacteroidete*s upon tRA treatments was positively correlated to proteinuria levels and glomerular pathological scores. These observations suggest that tRA induce similar changes in the gut microbiota regardless of the time of administration, and that these microbiota changes may exert both beneficial and detrimental roles.

Retinoids in combination with corticosteroids have been suggested as a potential therapy for SLE (11). However, our research group has shown that tRA has paradoxical effects on different tissue pathologies in genetically lupus-prone mice (12). tRA supplementation during the progressive phase of lupus mitigated glomerulonephritis but increased inflammatory cell infiltration to tissues other than the kidney. Additionally, we have shown that tRA given before lupus induction promotes the initiation of glomerulonephritis. Mechanistically, we have found that tRA treatment before lupus induction exacerbates the systemic and renal inflammation induced by pristane injection. It induces the activation of cDCs that can (1) enhance cell-mediated immunity by priming T_EM_ cells and (2) potentiate the activation of mesangial cells that in turn provide pro-fibrotic signals in the kidney. tRA supplementation during the progressive phase of lupus, on the other hand, does not augment pristane-induced activation of cDCs; instead, it exerts immunosuppressive functions that may benefit renal health. Furthermore, we have shown that both tRA treatments are able to modulate the microbial dysbiosis induced in the pristane model regardless of the time of administration. Collectively, our findings provide evidence for disease stage-dependent effects of tRA on the pathogenesis of SLE. As VA supplementation is a common practice, and people at risk for lupus may take it as well, further investigations are required to determine whether there is a need to recommend avoidance of extra VA for people genetically predisposed to develop lupus (e.g., first-degree relatives of SLE patients).

## Data Availability Statement

The datasets generated for this study can be found in the NCBI SRA database and Gene Expression Omnibus (GEO) repository under accession number GSE140104, as well as SRA database deposition number PRJNA605256.

## Ethics Statement

The animal study was reviewed and approved by IACUC at Virginia Tech.

## Author Contributions

XL conceived the study. XL and LA designed the experiments. LA performed the experiments. LA and SW analyzed the data. XC-P and BS contributed to mouse dissection and sample collection. JL, SL, and SS analyzed RNA-seq data. YL and AR measured retinol levels. TC and TL performed histopathological scoring. XL, LA, HW, and CR wrote the manuscript.

### Conflict of Interest

The authors declare that the research was conducted in the absence of any commercial or financial relationships that could be construed as a potential conflict of interest.
