# Supplementary material for: Retinoic Acid Exerts Disease Stage-Dependent Effects on Pristane-Induced Lupus
Source: Front Immunol. 2020 Mar 20;11:408. doi: 10.3389/fimmu.2020.00408 (PMC7103630; doi:10.3389/fimmu.2020.00408)
Supplement: Supplementary file 1 [file Data_Sheet_1.pdf]

Supplemental Information

Table S1. Primer sequences.

Target	Forward Sequence	Reverse Sequence
Tnfα	CCCTCACACTCAGATCATCTTCT	GCTACGACGTGGGCTACAG
Il1β	GCAACTGTTCCTGAACTCAACT	ATCTTTTGGGGTCCGTCAACT
Il18	GACTCTTGCGTCAACTTCAAGG	CAGGCTGTCTTTTGTCAACGA
Tgfβ1	CCACCTGCAAGACCATCGAC	CTGGCGAGCCTTAGTTTGGAC
Ccl2	TTAAAAACCTGGATCGGAACCAA	GCATTAGCTTCAGATTTACGGGT
Ccl3	TTCTCTGTACCATGACACTCTGC	CGTGGAATCTTCCGGCTGTAG
Ccl5	GCTGCTTTGCCTACCTCTCC	TCGAGTGACAAACACGACRGC
Laminin β1	AGACTTTGGGGGTTCATGTCA	ATCGTCCCGTCTCCTTGTCA
Itgal	CCAGACTTTTGCTACTGGGAC	GCTTGTTCGGCAGTGATAGAG
Occludin	TTGAAAGTCCACCTCCTTACAGA	CCGGATAAAAAGAGTACGCTGG
Claudin2	CAACTGGTGGGCTACATCCTA	CCCTTGGAAAAGCCAACCG
GAPDH	ATTGTCAGCAATGCATCCTG	ATGGACTGTGGTCATGAGCC
18S rRNA	GTAACCCGTTGAACCCCATT	CCATCCAATCGGTAGTAGCG

Table S2. tRA receptor binding sites (RAREs) found in the promoters of significant DEGs induced by tRA pre-treatment.

Gene	Gene ID	Gene position	Closest RARE position
S1pr1	ENSMUSG00000045092	chr3:115,413,351-115,417,973	chr3:115547044-115547543
Gpr146	ENSMUSG00000044197	chr5:139,856,535-139,872,369	chr5:139850830-139851329
Sox13	ENSMUSG00000070643	chr1:135,280,555-135,282,103	chr1:135276031-135276530<br>chr1:135294099-135294598<br>chr1:135305356-135305855<br>chr1:135329320-135329819<br>chr1:135331692-135332191<br>chr1:135264019-135264518
Alcam	ENSMUSG00000022636	chr16:52,268,274-52,452,578	chr16:52718347-52718846
Rfx2	ENSMUSG00000024206	chr17:56,915,320-56,970,431	chr17:56903748-56904247<br>chr17:56938686-56939185<br>chr17:56950556-56951055<br>chr17:56960331-56960830<br>chr17:56972757-56973256
Zfp677	ENSMUSG00000062743	chr17:21,520,712-21,536,229	chr17:21625553-21626052
Riken/ Scart2	ENSMUSG00000054672	chr7:147,433,277-147,485,690	chr7:147417028-147417527<br>chr7:147416877-147417376<br>chr7:147416592-147417091
Nesh	ENSMUSG00000035258	chr16:56,477,959-56,690,241	chr16:56868421-56868920
Tnfrsf26	ENSMUSG00000045362	chr7:150,793,568-150,813,858	chr7:150358888-150359387
Arl4c	ENSMUSG00000049866	chr1:90,569,700-90,598,451	chr1:90574823-90575322<br>chr1:90565337-90565836
Banp	ENSMUSG00000025316	chr8:124,473,650-124,521,083	chr8:124490956-124491455<br>chr8:124490763-124491262<br>chr8:124472627-124473126
Pde3b	ENSMUSG00000030671	chr7:121,558,795-121,682,765	chr7:121277355-121277854
Atp2b1	ENSMUSG00000019943	chr10:98,377,786-98,488,777	chr10:98337763-98338262
Camp	ENSMUSG00000038357	chr9:109,749,889-109,751,970	chr9:109781968-109782467
Ngp	ENSMUSG00000032484	chr9:110,322,254-110,325,514	chr9:110251478-110251977<br>chr9:110394017-110394516
Ltf	ENSMUSG00000032496	chr9:110,921,796-110,945,270	chr9:110862675-110863174
Tnfrsf26	ENSMUSG00000045362	chr7:150,793,568-150,813,858	chr7:150358888-150359387
Arhgap24	ENSMUSG00000057315	chr5:103,274,026-103,326,848	chr5:103620457-103620956
Ndufaf8	ENSMUSG00000078572	chr11:119,960,242 -119,962,018	chr11:119935419-119935918
Rpl36	ENSMUSG00000057863	chr17:56,752,818-56,753,666	chr17:56857196-56857695

Table S3. Significantly modulated bacterial species within the phyla *Bacteroidetes* and *Firmicutes* upon pristane induction of lupus and tRA treatments.

**Bacterial species**	**Effect**	**Start of modulation (weeks post induction)**	**Groups**
Bacteroides_thetaiotaomicron	Increased	4	tRA-Post.
Muribaculum_intestinale	Increased	10	tRA-Post.
Muribaculum_intestinale	Increased	20	tRA-Pre.
Odoribacter_splanchnicu	Increased	2	tRA-Pre.
Odoribacter_splanchnicu	Increased	12	tRA-Post.
Alistipes_finegoldii	Increased	6	tRA-Pre.
Alistipes_finegoldii	Increased	12	tRA-Pre.
Alistipes_onderdonkii	Increased	2	tRA-Pre.
Alistipes_shahii	Increased	2	tRA-Post.
Alistipes_shahii	Increased	22	tRA-Pre.
Enterococcus_dispar	Increased	2	tRA-Post.
Lactobacillus_gasseri	Decreased	2	Pristane-alone & tRA-Pre/Post.
Mordavella_massiliensis	Decreased	4	Pristane-alone & tRA-Pre.
Mordavella_massiliensis	Decreased	6	Pristane-alone & tRA-Pre/Post.
Clostridium_aldenense	Increased	2	tRA-Post.
Clostridium_hylemonae	Decreased	2	tRA-Pre/Post.
Clostridium_lactatifermentans	Increased	2	tRA-Pre.
Clostridium_methylpentosum	Increased	2	tRA-Pre.
Clostridium_saccharogumia	Increased	6	tRA-Pre.
Clostridium_scindens	Increased	4	tRA-Pre.
Blautia_hydrogenotrophica	Decreased	12	tRA-Pre/Post.
Butyrivibrio_crossotus	Decreased	6	tRA-Post.
Eisenbergiella_tayi	Increased	10	tRA-Post.
Eubacterium_coprostanoligenes	Increased	6	tRA-Post.
Eubacterium_oxidoreducens	Increased	2	Pristane-alone
Eubacterium_siraeum	Increased	2	tRA-Post.
Frisingicoccus_caecimuris	Increased	6	tRA-Post.
Marvinbryantia_formatexigens	Increased	6	tRA-Pre.
Roseburia_faecis	Decreased	4	tRA-Pre/Post.
Ruminococcus_champanellensis	Increased	6	Pristane-alone
Acutalibacter_muris	Decreased	2	Pristane-alone & tRA-Post.
Butyricicoccus_pullicaecorum	Decreased	10	tRA-Post.
Intestinimonas_butyriciproducens	Increased	4	tRA-Post.
Neglecta_timonensis	Increased	10	tRA-Pre.
Oscillibacter_ruminantium	Increased	2	tRA-Pre.
Oscillibacter_valericigenes	Increased	10	tRA-Pre.
Phocea_massiliensis	Decreased	4	tRA-Pre/Post.
Pseudoflavonifractor_phocaeens	Decreased	8	tRA-Pre/Post.

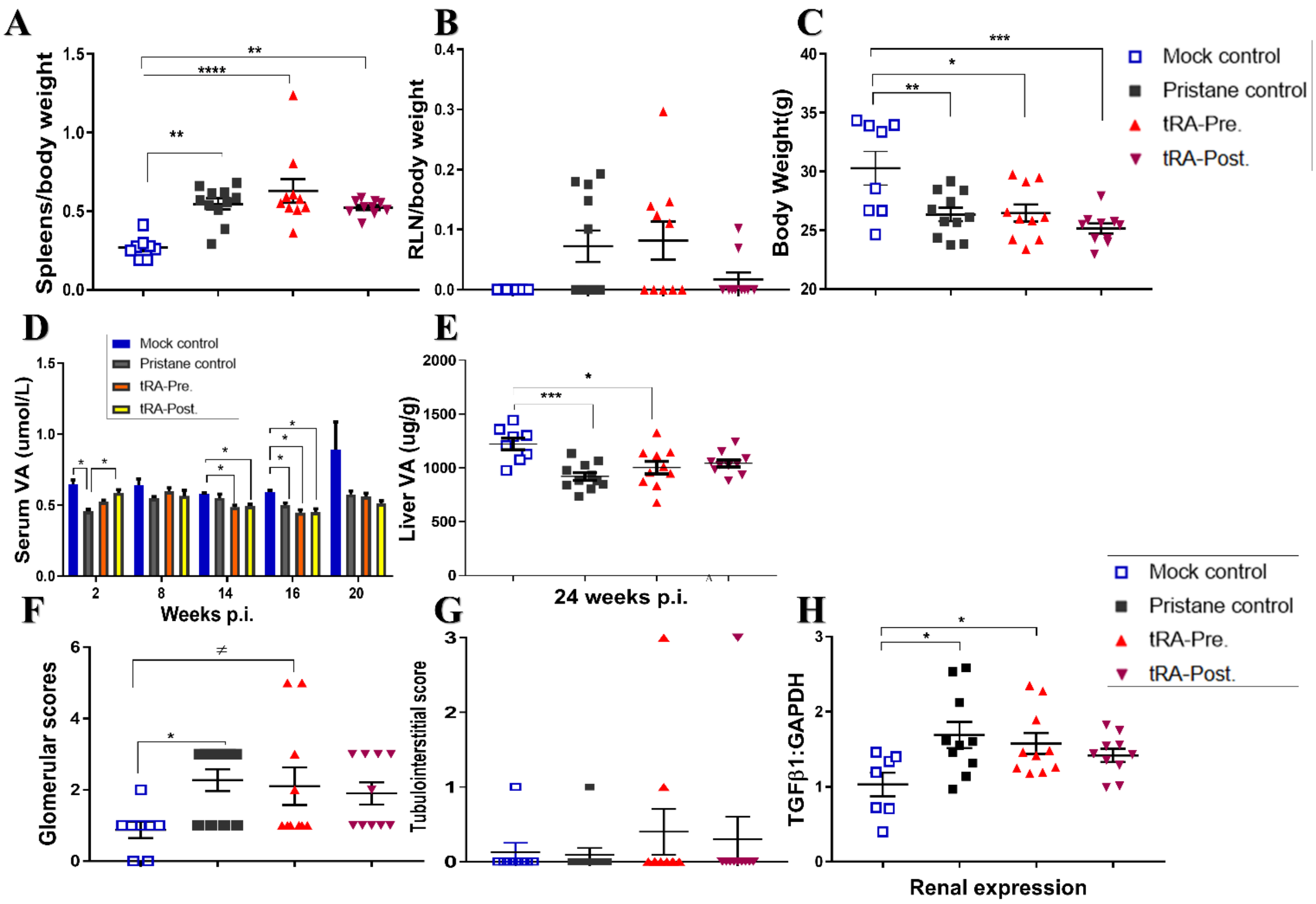

Figure S1. (A) Splenomegaly shown as spleen to body weight ratios. (B) Enlarged RLNs shown as weight ratios. (C) Final body weight in grams at 6 months post pristane injection. (D) Serum levels of retinol/VA over time starting 2 weeks post lupus induction. Data were obtained from pooled serum samples (n>8 per group per time point) as quantified with UPLC. (E) Concentrations of retinol/VA in snap-frozen liver tissues at the experimental endpoint. (F) Composite glomerular histopathological scores (#P = 0.0816). (G) Tubulointerstitial scores. (H) Renal transcript level of *Tgfβ1* after normalization to that of GAPDH.

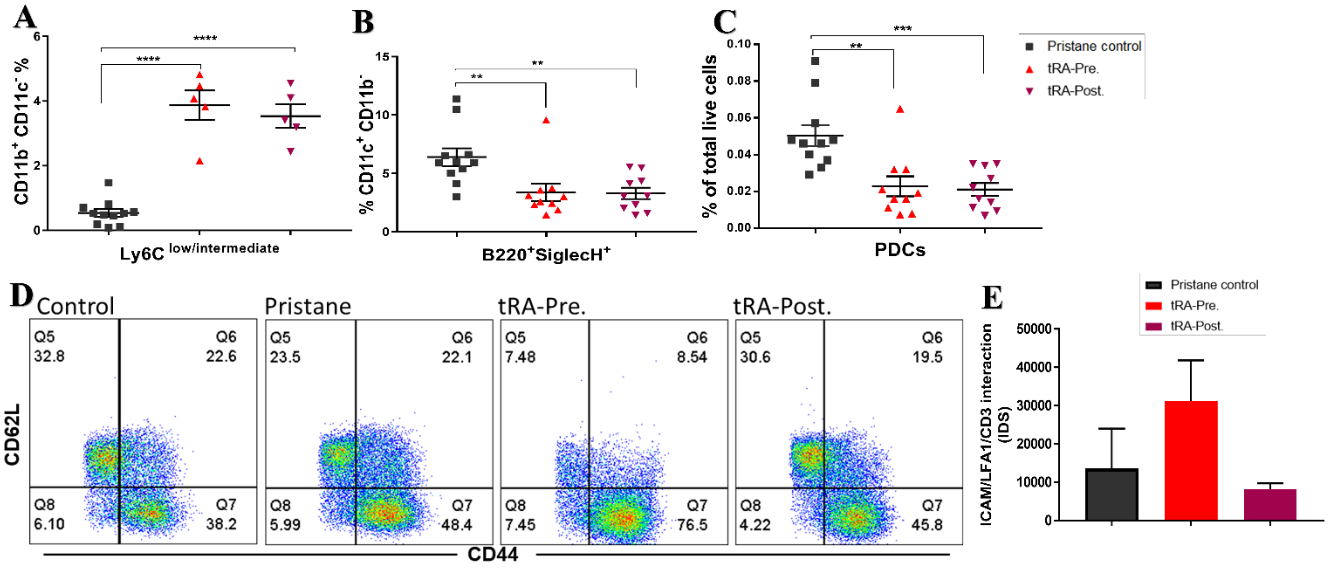

Figure S2. (A) Percentage of MDSCs gated as Ly6C$^{low/intermediate}$ CD11b^{+}CD11c^{-}. (B) B220^{+}Siglec-H^{+} pDCs as the percentage of CD11c^{+}CD11b^{-} DCs. (C) Percentage of pDCs gated as CD11c^{+}CD11b^{-} B220^{+}Siglec-H^{+}. (D) Representative flow cytometry plots showing the percentages of splenic CD62L^{+}CD44^{-} naïve T cells *vs.* CD62L^{-}CD44^{+} T_{EM} cells gated on CD3^{+} T cells. (E) The integrated density scores (IDS) calculated with ImageJ for co-localization of CD3, CD11/CD18 (or LFA) and CD54 (or ICAM1) in immunohistochemically stained splenic sections. While trending, the tRA-treated groups were not significantly different from the pristane control.

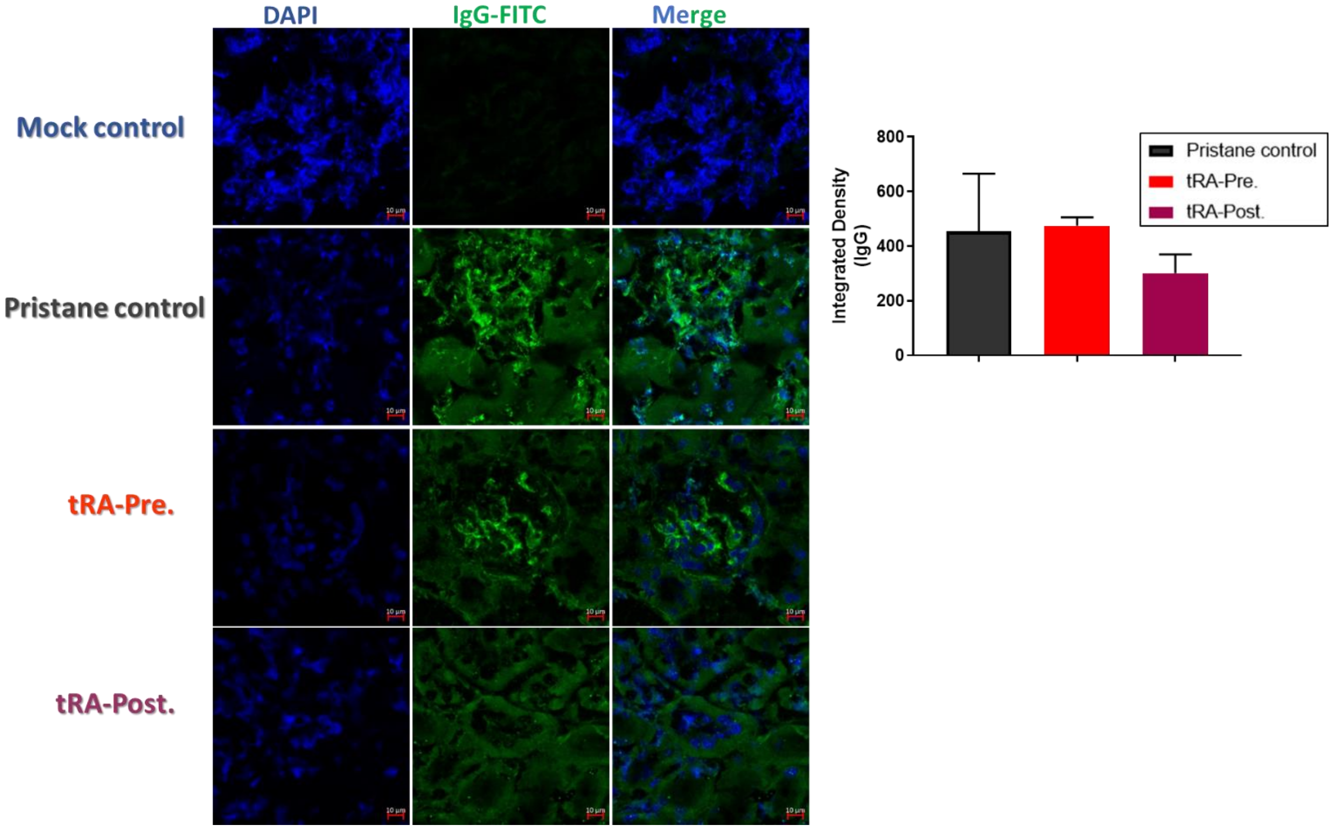

Figure S3. IgG deposition in the kidney as determined with immunohistochemical staining at the experimental endpoint. DAPI (left), IgG-FITC (middle) and merged micrographs (right) are shown. Images were captured with a Zeiss LSM 880 confocal microscope. Integrated density scores for the IgG-FITC fluorescence were calculated using ImageJ.

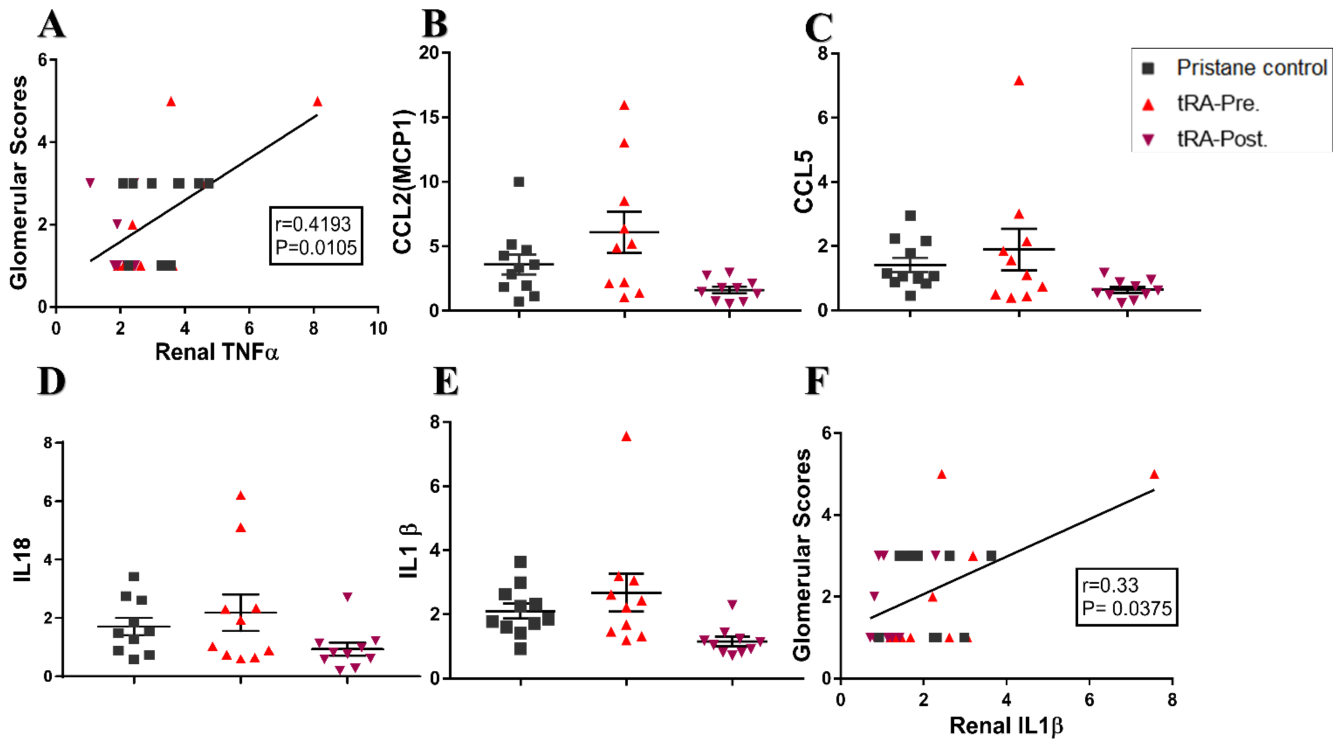

Figure S4. (A) Correlation between glomerular pathological scores and the renal expression of *Tnfα*. (B-E) Renal transcript levels of *Ccl2*/*Mcp1* (B), *Ccl5* (C), *Il18* (D) and *Il1β* (E) as determined with RT-qPCR. (F) Correlation between glomerular pathological scores and the renal expression of *Il1β*.

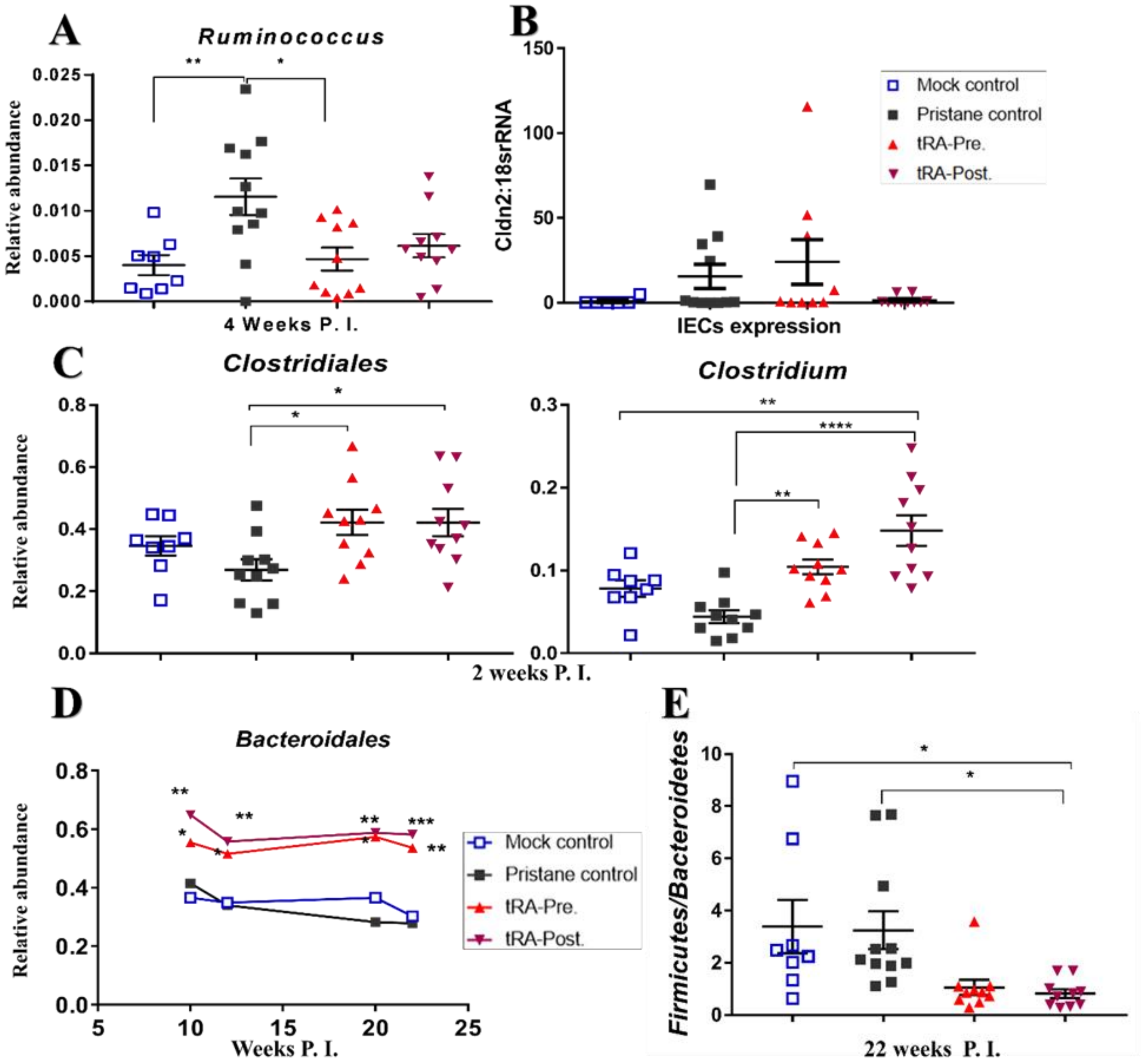

Figure S5. (A) Relative abundance of the genus *Ruminococcus* determined with 16S rRNA sequencing at 4 weeks post lupus induction. (B) Transcript level of Claudin-2 (*Cldn2*) normalized to that of 18S rRNA in IECs as determined with RT-qPCR. (C) Relative abundance of *Clostridiales* and genus *Clostidridium* at 2 weeks post pristane injection. (D) Relative abundance of *Bacteroidales* over time. (E) Ratio of *Firmicutes/Bacteroidetes* at 22 weeks post lupus induction.
